# Origin of the Functional Distinctiveness of NF-κB/p52

**DOI:** 10.3389/fcell.2021.764164

**Published:** 2021-11-23

**Authors:** Gourisankar Ghosh, Vivien Ya-Fan Wang

**Affiliations:** ^1^ Department of Biochemistry, University of California, San Diego, San Diego, CA, United States; ^2^ Faculty of Health Sciences, University of Macau, Taipa, Macau SAR, China; ^3^ Cancer Centre, Faculty of Health Sciences, University of Macau, Taipa, Macau SAR, China

**Keywords:** NF-κB, p52, transcriptional regulation, DNA binding, diseases

## Abstract

The transcription regulators of the NF-κB family have emerged as a critical factor affecting the function of various adult tissues. The NF-κB family transcription factors are homo- and heterodimers made up of five monomers (p50, p52, RelA, cRel and RelB). The family is distinguished by sequence homology in their DNA binding and dimerization domains, which enables them to bind similar DNA response elements and participate in similar biological programs through transcriptional activation and repression of hundreds of genes. Even though the family members are closely related in terms of sequence and function, they all display distinct activities. In this review, we discuss the sequence characteristics, protein and DNA interactions, and pathogenic involvement of one member of family, NF-κB/p52, relative to that of other members. We pinpoint the small sequence variations within the conserved region that are mostly responsible for its distinct functional properties.

## Discovery and Early Description of p52/NF-κB2

The founding member of the NF-κB family is cRel (Chen et al., 1983; Wilhelmsen et al., 1984). The cellular function of cRel protein was unknown at the time of the *cRel* gene discovery. The biochemical characterization of a factor that triggers the production of the kappa light chain gene by binding to the enhancer element led to the discovery of the classical NF-κB factor, the p50:RelA heterodimer (Sen and Baltimore, 1986; Baeuerle and Baltimore, 1988). It was clear at that point that cRel, which bears strong sequence homology to both p50 and RelA (formerly p65) would also operate as a transcription factor (Ghosh et al., 1990; Nolan et al., 1991; Ruben et al., 1991). The primary structure of the gene which encoded p50, however, was distinct from RelA and cRel since it encoded a 105 kDa protein instead of a 50 kDa protein (Bours et al., 1990; Meyer et al., 1991). The C-terminal half of p105 is degraded by proteasomes yielding the p50 protein (Blank et al., 1992; Liou et al., 1992). Two groups independently discovered p52, the fourth member of the family (Neri et al., 1991; Schmid et al., 1991). DNA sequence homology-based screening of cDNA libraries identified a gene encoding a 100 kDa protein with strong primary sequence and structural homology with p105 (Bours et al., 1992; Mercurio et al., 1992). Regulated processing of p100 yields the DNA binding transcription factor, p52. Another group studying B cell lymphoma showed that chromosomal fusion generated a transcript at the fusion locus that encodes a protein with strong sequence similarity to p50. As expected, in normal cells the gene encoded a 100 kDa protein which the discoverers named Lyt-10 (Neri et al., 1991). C-terminal truncations of the NF-κB2/p100 have been observed in a number of cases of B and T cell lymphomas, and myelomas. The C-terminal halves of both p105 and p100 act as inhibitors of the N-terminal DNA binding domain as well as other NF-κB proteins. It subsequently became clear that abolishment of the inhibitory activity and constitutive DNA binding activity of p52 due to the truncation of the C-terminal domain contributes to malignancies.

## The NF-κB Family

**FIGURE 1:**
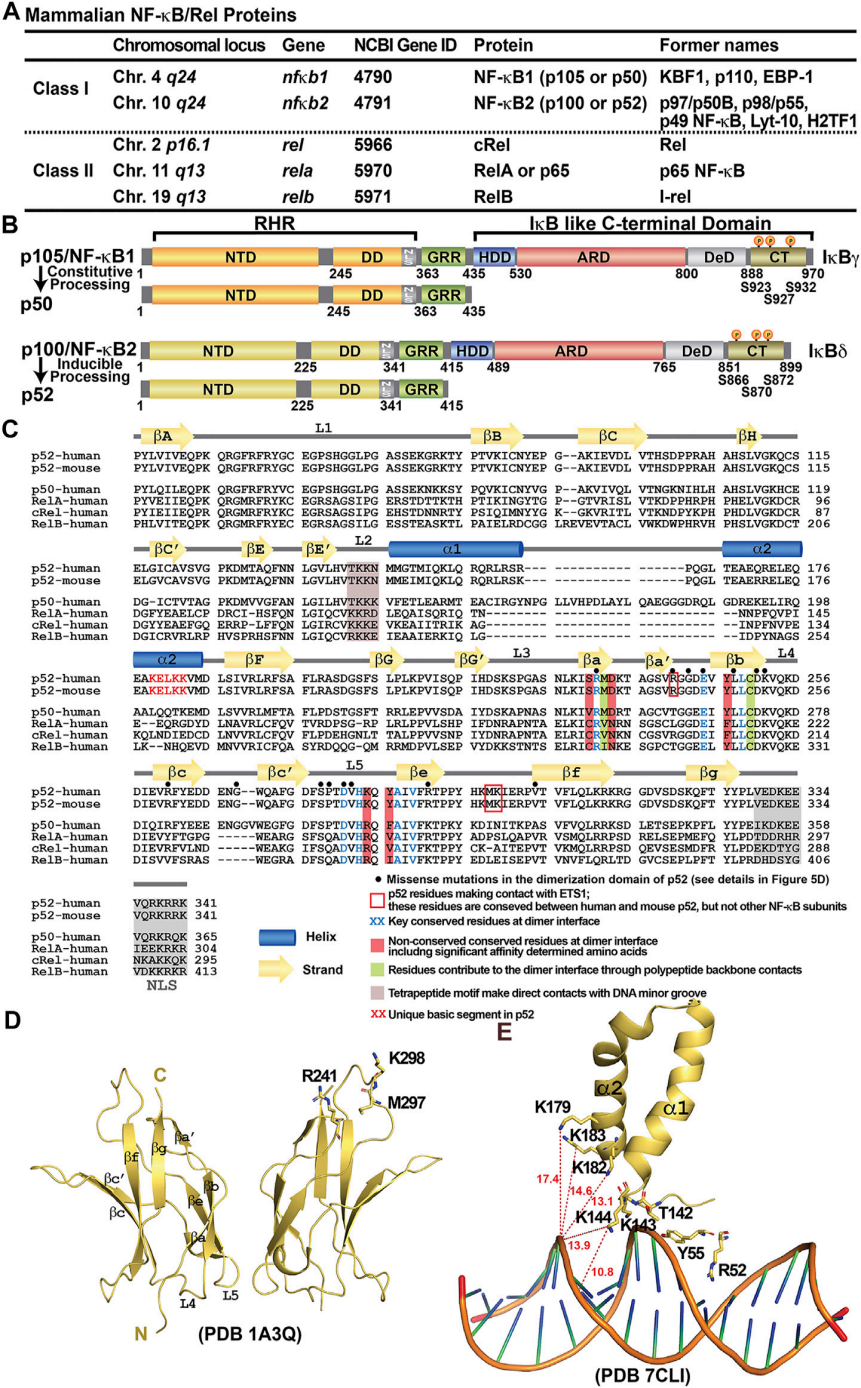
Discovery of NF-κB and NF-κB dimers. **(A)** Table summarizing the mammalian NF-κB proteins. The chromosomal locus on the human genome, the gene name and the Gene ID at NCBI of the five family members are listed. Due to the complexity of the family at both genetic and biochemical levels, these proteins have been named with various nomenclature throughout their discoveries; their former names in the literature were also indicated in the table. **(B)** Domain organization of class I NF-κB: NF-κB1 (p105/p50) and NF-κB2 (p100/p52). **(C)** Sequence alignment of the RHR of NF-κB subunits. Both human and mouse sequences of the p52 subunit are shown. Only human sequences are shown for the rest of the family members. Secondary structures and connecting loops are drawn above the sequences. The NLS is highlighted in grey. Black circles on top of the sequences indicates the missense mutations in the dimerization domain of p52, detail information is listed in Figure 5D. **(D)** Ribbon diagram of the p52 DD homodimer indicating the secondary structure elements and overall immunoglobulin (Ig)-like fold. Three residues interacting with ETS1 are indicated; they are located away from the dimer interface. These residues are conserved between mouse and human p52, but not present in other NF-κB subunits. **(E)** A unique basic segment in p52 NTD helix α2 interacts with target DNA.

The fifth NF-κB protein, RelB, was discovered using homology-based screening approaches (Ruben et al., 1992; Ryseck et al., 1992; Bours et al., 1994). With the inclusion of RelB, the mammalian NF-κB family containing only five members was complete. The five NF-κB proteins can be further divided into two sub-classes: the p50 and p52 subunits belong to class I by virtue of their lack of a transcriptional activation domain (TAD). Instead of a conventional TAD, p50 and p52 have a brief ∼70-residue segment rich in glycine residues at their C-termini, named glycine rich region (GRR). The other three subunits, RelA, cRel and RelB, constitute class II and each contains a TAD at their C-termini (Figure 1A). The N-termini of all family members share a high degree of sequence homology known as the Rel Homology Region (RHR) (Figures 1B,C). This roughly 300 residues long segment is responsible for site-specific DNA binding, combinatorial dimer formation and inhibitor of NF-κB (IκB) protein binding. As predicted from the high sequence similarity, their folded structures are also highly conserved. The RHR is separately folded into two domains; the N-terminal domain (NTD), consisting of nearly 200 residues, is primarily responsible for sequence-specific DNA binding, and the C-terminal dimerization domain (DD) (Figures 1C–E). A short 10-residue linker connects these two folded domains (Ghosh et al., 2012). The dimers are formed by β-sheet sandwich between the two Ig-like fold of the DD in a symmetrical manner (Figure 1D). Only a small pool of NF-κB dimers is present in the nucleus under homeostatic conditions to maintain transcription of a limited number of genes (Tergaonkar et al., 2005; O’Dea et al., 2007; Mathes et al., 2008). A greater pool of NF-κB dimers (or monomers) remains inactive, bound to a class of inhibitor protein known as IκB. The DD and a short peptide C-terminus to the DD, called nuclear localization signal (NLS), which caps the RHR, are the binding site of IκB (Huxford et al., 1998; Jacobs and Harrison, 1998; Malek et al., 1998; Bergqvist et al., 2008). Different IκB proteins interact with selective NF-κB dimers and they all play a role in NF-κB signaling. Among IκB proteins, IκBα was the first one to be identified and cloned and is the best characterized inhibitor of NF-κB. The IκBα-bound NF-κB complexes are inactive even when they are in the nucleus (Bergqvist et al., 2009; Dembinski et al., 2017). In response to inflammatory and pathogenic stimuli such as tumor necrosis factor-alpha (TNFα) and lipopolysaccharide (LPS), IκBα is phosphorylated at S32 and S36 by the IκB kinase (IKK) complex (Brown et al., 1995; Traenckner et al., 1995; DiDonato et al., 1996). Phosphorylation leads to E3 ligase mediated polyubiquitination and rapid degradation through the 26S proteasomal pathway (Alkalay et al., 1995; Chen et al., 1995; Lin et al., 1995; Scherer et al., 1995; Baldi et al., 1996; Yaron et al., 1998). Therefore, the amount of active pool of NF-κB dimers is significantly enhanced when cells are stimulated.

Many researchers have been investigating the dimeric NF-κB family because of its critical roles in a range of biological processes, most important of which is the control of inflammation (Karin and Greten, 2005; Lawrence, 2009; Sun, 2017; Zhang et al., 2017). NF-κB dimers regulate hundreds of effector genes that collectively govern inflammation and other cellular activities. Several reviews have been written about processes by which NF-κB regulate cell physiology. The focus of this review is on p52, its DNA binding as a homodimer, its cofactor functions, interactions with its coregulators Bcl3 and RelB, and its role in pathogenesis from the viewpoint of biochemical mechanisms. We hope this will alert others working on the biological front to obtain useful information to understand biological regulations.

## Generation of p52/NF-κB2 From p100

**FIGURE 2:**
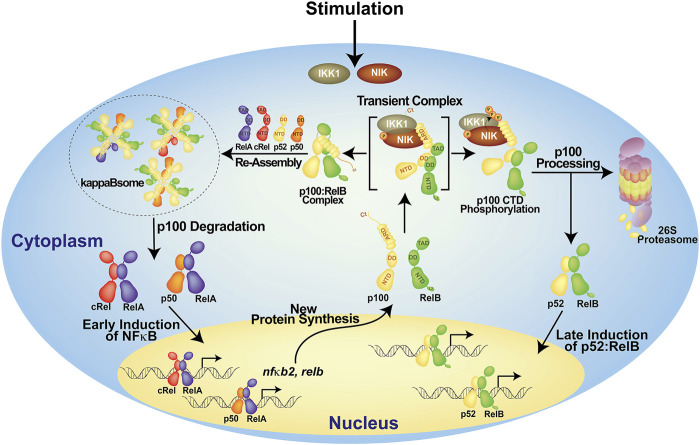
A model of the generation of p52 from p100. In the resting cells, p100 forms high molecular weight complexes known as kappaBsomes; it inhibits nearly all NF-κB subunits. Upon stimulation, p100 will be degraded resulting in the early induction of NF-κB which in term activates *nfκb2* gene itself. The newly synthesized p100 protein gets phosphorylated at the C-terminal serine residues, then it is ubiquinated and processed into p52 which heterodimer with RelB and regulate its target genes. Other possible modes of p100 processing are not shown here.

No review on p52 can be complete without the discussion of its production from the precursor protein, p100. Despite its origin as the precursor of p52, p100 has been shown to be an essential protein in its own right (Novack et al., 2003; Basak et al., 2007; Sun, 2017). Although p100 is a member of the IκB family protein, it functions differently than the classical IκBs, IκBα-, β- and -ε. When p100 is left unprocessed, it forms high molecular weight complexes known as kappaBsomes (Tao et al., 2014; Mitchell et al., 2016). In these complexes, p100 interacts with other NF-κB proteins via both its N-terminal p52 domain and its C-terminal IκB-like domain. Four p100 molecules form a tetrameric complex through a segment called helical dimerization domain (HDD) which connecting the two halves, wherein four N-terminal p52 domains can engage four other monomers, including processed p52, within the family (Savinova et al., 2009; Tao et al., 2014). RelB was proven to be an essential binding partner in these complexes. Two p52 monomeric segments are likely to attach to two RelB subunits whereas two other p52 monomeric segments could bind to RelA and cRel in any combinations. And four C-terminal IκB-like domains can interact with four dimers. Because of its ability to interact with four separate members of the NF-κB family, p100 is a one-of-a-kind inhibitor. Indeed, in unstimulated cells, p100 inhibits a considerable portion of total NF-κB proteins (Figure 2). Unprocessed p105 is also an inhibitor of NF-κB. However, p105 only forms a dimer, unlike p100, inhibits only two NF-κB family members (Savinova et al., 2009). Furthermore, unlike p100, p105 does not inhibit p52 or RelB, making it a restricted inhibitor. Heterocomplexes between p100 and p105 also exist. Whether such a complex assembles as a dimer, tetramer or even larger oligomer is not known (Yılmaz et al., 2014).

The phosphorylation of p100 at two to three serine residues located near the C-terminus by two protein kinases, NF-κB inducing kinase (NIK) and IkappaB kinase 1 (IKK1) (also known as IKKα), is required for the conversion of p100 into p52 (Senftleben et al., 2001; Xiao et al., 2001; Fong and Sun, 2002; Liang et al., 2006). After then, phospho-p100 is subsequently subjected to ubiquitination and degradation by the 26S proteasome (Sun, 2017). Only newly synthesized p100, but not pre-existing p100, can be processed (Mordmüller et al., 2003; Yılmaz et al., 2014). The pre-existing p100 molecules are integral part of the kappaBsomes that are protected from being processed (Figure 2). It is proposed that the proteasome has two activities: it completely destroys p100 using one activity and promotes processing with the other activity through endoproteolytic cleavage followed by selective degradation of the C-terminus. In kappaBsomes, the site of p100 processing is not free whereas in newly synthesized p100 the site remains open before it is masked by NF-κB subunits towards forming the kappaBsomes. It has been proposed that the production of the appropriate amount of p52 from p100 occurs during the assembly of the kappaBsomes, and the competition between RelB and NIK for p100 dictates the ratio of p52 to p100 (Fusco et al., 2016).

Yilmaz et al. showed that lymphotoxin beta (LTβ), a stimulus of the non-canonical signaling pathway, can induce simultenaous processing of p100 and p105 possibly from the p105/p100 heterocomplexes. In a recent publication, Chawla et al. also demonstrated simultaneous processing of p105 and p100 through non-canonical signaling generating p50:RelA and p52:RelA complexes in intestinal epithelial cells (Chawla et al., 2021). Thus, a variety of NF-κB dimers can arise from the processing of p105, p100 and p105/p100 kappaBsomes in a stimulus specific manner.

A footnote here about the nomenclature; IκBδ and IκBγ refer to the inhibitory biochemical activities of p100 and p105, respectively (Rice et al., 1992; Basak et al., 2007; Savinova et al., 2009). However, researchers frequently refer to these two proteins’ IκB-like C-terminal domains as IκBδ and IκBγ resulting in a muddled nomenclature.

## Preferential p52 Dimers

**FIGURE 3:**
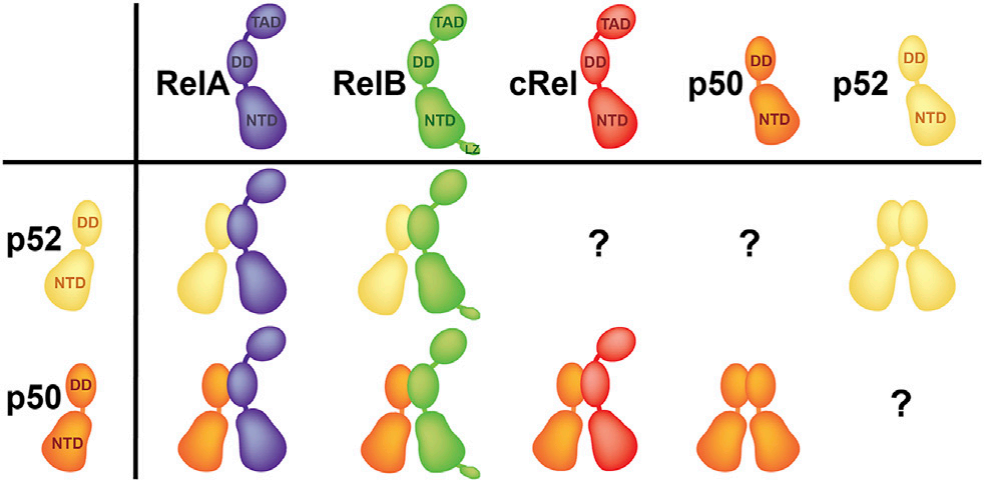
Preferential dimers. p52:RelB and p52:RelA heterodimers, as well as the p52:p52 homodimer are the three p52 dimers have been seen in cells. p50 is the only subunit which forms four out of five possible dimers.

In theory, p52 should form five dimers; four heterodimers with four other family members and the p52:p52 homodimer. The p52:RelB and p52:RelA heterodimers, as well as the p52:p52 homodimer are the three most abundant p52 dimers *in vivo* (Basak et al., 2008; Smale, 2012) (Figure 3). Although p52 may form dimers with p50 and cRel, these dimers are not observed in cells. The timing and tissue restricted expression of p50, p52 and cRel could be incompatible with their dimer formation. The more likely explanation is that the relative strength of the dimers and the concentration of accessible subunits differ, resulting in a graded dimer formation process that ranges from no dimers to highly stable dimers. As a result, the p50:p52 and p52:cRel heterodimers are likely to be less stable than the other p52 dimers found in cells. The crystal structures of several NF-κB homo- and heterodimers are known. With the exception of the non-physiological RelB homodimer, these dimers are very similar in appearance and are formed by a highly conserved set of interfacial residues (Ghosh et al., 1995; Muller et al., 1995; Cramer et al., 1997; Chen F. E. et al., 1998; Chen Y.-Q. et al., 1998; Huang et al., 2005; Moorthy et al., 2007; Fusco et al., 2009). Therefore, visualization of the dimer interface cannot appropriately explain why some of the NF-κB dimers are not seen *in vivo*. Small variations at the interfacial residues allow us to speculate why some dimers are stronger than others, but we cannot explain why p52 is unable to dimerize with p50 and cRel or the preferential formation of the p52:RelB, p52:RelA and p52:p52 dimers. This puzzle only points to residues outside of the dimer interface as predictors of dimer stability or preferred dimer formation. Consistent with this notion, it was reported that different segments of RelB DD contribute differentially toward heterodimerization with p50 and p52. Several of these residues are located outside of the dimer interface (Ryseck et al., 1995). Vu and colleagues demonstrated that converting all interfacial residues of RelB into RelA or p50 did not result in a stable RelB homodimer implying that non-interfacial residues play an important role in dimer stability (Vu et al., 2013). However, it is unknown how sequences outside of the dimer interface regulate dimerization ability. Although the DDs of p50 and p52 are 68% identical and 83% homologous in primary sequence, the p50 DD is well behaved in solution, which appears to be more stable and less aggregation prone than the p52 DD. Eleven of the fourteen residues that make up the dimer interface are identical between p50 and p52, while two are homologous (R to K and F to Y changes from p50 to p52 at corresponding positions) (Figure 1C). R305A mutant of p50 revealed that the side chain has very little contribution to the dimer stability, while the F307 (p50) to Y change is unlikely to have any effect because only the benzene ring is involved in the interface formation with no role for the -OH group (Sengchanthalangsy et al., 1999). Indeed, these two homologous residues of p50 and p52 homodimers make identical cross subunit van der Waals interactions (Huang et al., 1997; Vu et al., 2013). It is worth noting that p50 is the only member of the NF-κB family that makes four of the five possible dimers (Figure 3). What makes p50 so adaptable to receiving the other monomers forming biological dimers needs more investigation. A detailed comparison of the structures of the dimerization domains of p50 and p52 reveals no visible differences. A few differences in the loop regions of the two subunits appears innocuous and do not seem to cause any effect. It appears that the p52 DD has a more stable hydrophobic core than the p50 DD, implying that the adaptable core of p50 DD permits the interfacial residues to adjust to better interactions with the opposing subunit, forming a more stable homodimer. This suggests that the energetics of all core residues collectively influence the properties of surface residues, rather than a single or a pair of residues being responsible for imparting surface features. To provide a fuller picture of the differences, more detailed future investigations across all dimers are needed.

In addition to the protein sequence-driven stability differences among the NF-κB dimers, other factors could also be playing critical roles, such as competition with other subunits and/or inhibitors. In the competing environment *in vivo*, RelA primarily exists as the p50:RelA heterodimer in activated cells or as an inhibited IκBα:p50:RelA complex in resting cells. Therefore, RelA does not have much of a chance to associate with p52. It is also worth noting that while RelA is not inducibility expressed, it induces the expression of p100/p52 and RelB. This spaciotemporal modulation increases the likelihood of formation of the p52:RelB dimer over the p52:RelA heterodimer.

## Transcriptional Regulation by DNA Binding

**FIGURE 4:**
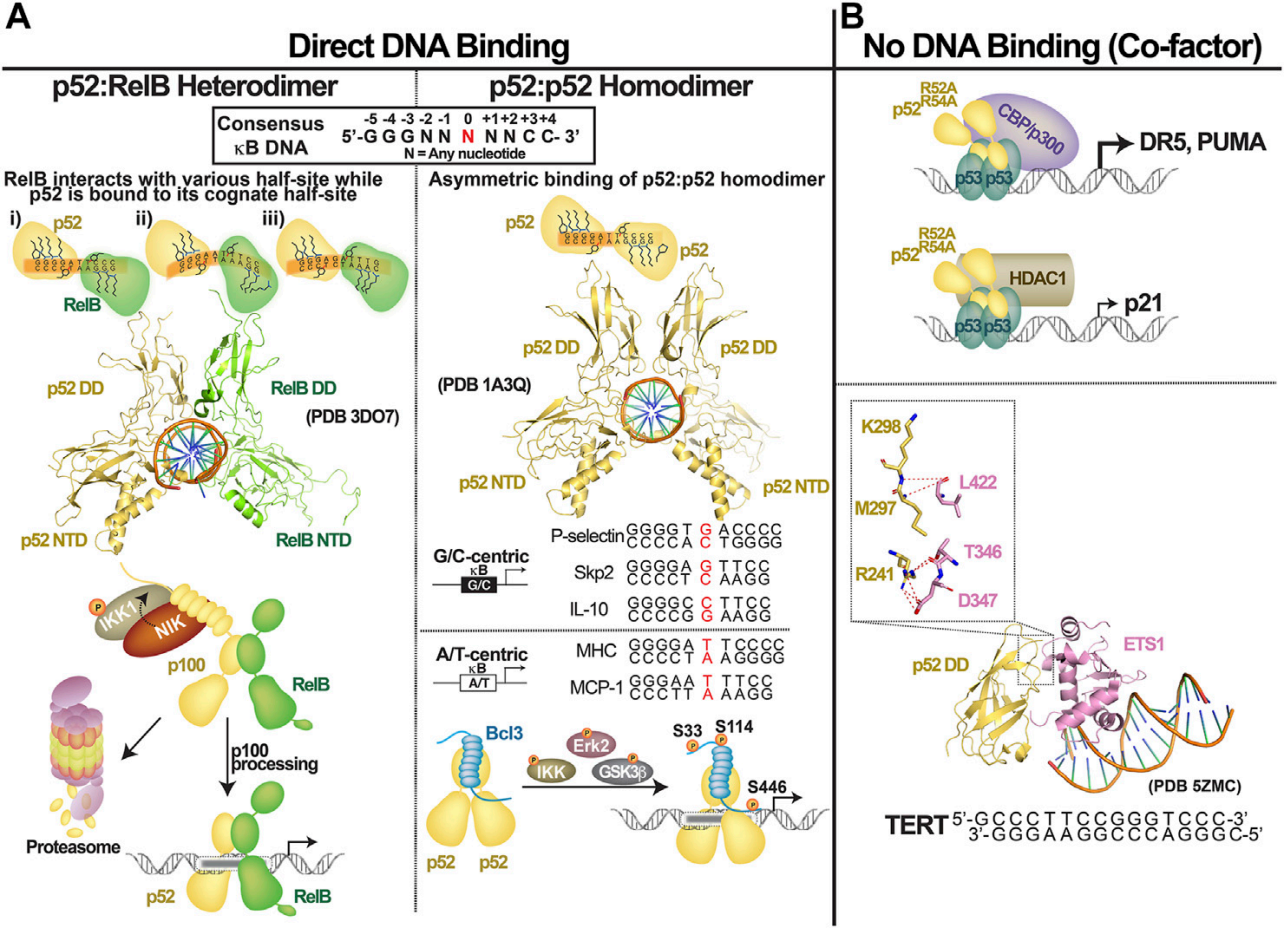
Transcriptional regulation by NF-κB2/p52. p52 regulates gene expression by: **(A)** direct binding to the consensus κB DNAs as **(left)** p52:RelB heterodimer or **(right)** p52:p52 homodimer which functions together with Bcl3; **(B)** p52 could also function as co-factor by interacting with other transcription factors such as p52 and ETS1.

The structure of the p52 homodimer bound to a κB DNA is known. Basic features of the p52:p52:MHC-κB DNA complex are similar to those of the p50:p50:MHC-κB-like DNA complex (Muller et al., 1995; Cramer et al., 1997; Chen F. E. et al., 1998). MHC-κB DNA has an 11-bp consensus sequence where two 5 bp half sites are split at the center by an A/T-bp at the center (A/T-centric κB site). The p52:p52 homodimer binds two symmetrical half-sites asymmetrically, as do all NF-κB dimers. A p52 protomer contacts all 5 bp directly at one half-sites, while the second protomer does not directly contact the last bp at the other half-site (Figure 4A, right). This shows that NF-κB protomers perceive DNA sites differently even when the κB DNA is virtually flawless. Since κB DNA sequences vary widely with some extremes having only a half-site consensus, the binding modalities of two protomers are expected to be dramatically different. The biological significance of sequence variations is only beginning to be clarified. Most κB DNAs identified showed NF-κB dimers bind to consensus DNA containing A/T at the central position (A/T-centric) simply because these sites are the preferred sites for the most widely studied p50:RelA heterodimer (Chen and Ghosh, 1999). The p52 homodimer has been shown to bind both an A/T- and a G/C-centric site with similar affinities but with different functional outcomes. It activates transcription by binding to G/C-centric sites but represses transcription by binding to A/T-centric sites (Wang V. Y.-F. et al., 2012). Some of the key genes activated by the p52:Bcl3 homodimer complex include P-selectin, Skp2 and Cyclin D1. They all contain a G/C-centric κB site. Under the condition where p52 or Bcl3 is overexpressed high expression of Skp2 and Cyclin D1 results in unchecked proliferation as seen in many cancers (Guttridge et al., 1999; Barre and Perkins, 2007). Most NF-κB-regulated promoters contain multiple κB sites, often having both G/C- and A/T-centric κB sites. The p52:Bcl3 complex is expected to mediate mixed regulation of these sites controlling the overall levels of transcripts.

The p50:RelA heterodimer or the p50 and RelA homodimers bind G/C-centric sites with significantly lower affinity. Because the central bp does not directly contact the dimers, it is most likely that this bp allosterically affects the binding of NF-κB dimers to the κB DNAs. The p52 homodimer is unique in this regard since it binds both A/T- and G/C-centric DNA. Indeed, we found that A/T- and G/C-centric κB or κB-like DNAs are structurally distinct (PDB accession number 7CLI). The G/C-centric DNAs have a substantially wider minor groove than the A/T-centric DNAs. p52 has the ability to recognize structurally distinct κB DNAs, whereas p50 and RelA do not. Interestingly, p52 binds to these two classes of κB DNA by taking two separate routes: using entropy to bind G/C DNA and using enthalpy to bind A/T DNA.

What sets p52 apart from the rest of the family? A tetrapeptide motif placed near the minor groove is critical for DNA binding by all NF-κB members for DNA binding. Except p52, which contains two basic residues, others contain three (p50: T^142^KKK; p52: TKKN; RelA: KKRD) (Figure 1C). The central ‘KK’ dipeptide motif makes direct contact with the DNA at the minor groove. The basic residue absent in p52 mediates long range electrostatic interaction with the DNA phosphate backbone at the inner core of κB DNA in other subunits. Interestingly, p52 has a unique basic segment that is not found in other members (K^179^ELKK in p52 versus VQQTK in p50). This basic segment is destined to have an effect on how p52 interacts with the target DNA (Figures 1C,E). We propose that these sequence differences explain discriminatory DNA interactions by the NF-κB dimers, including variations in the central core region of κB DNAs. We are tempted to speculate that these unique sequence characteristics enable p52 to bind both A/T- and G/C-centric κB sites near equal affinities while employing different binding strategies. These changes in protein sequence could also explain why the p50 and p52 homodimers, which recognize most κB sites with similar affinities, diverge in a few cases (Schmid et al., 1994). For instance, the GGAACGTCCC DNA binds five times more tightly to the p52 homodimer while the GGGGGCTCCC DNA binds significantly tighter to the p50 homodimer (Udalova et al., 2002; Nijnik et al., 2003). The first DNA is quite similar to the Ig-κB DNA with the exception of 1 bp difference in the middle, GGGAC G TTCC (the central bp is in red color). The central G/C base pair is discriminated against by the p50 homodimer, and as described above, p52 binds to both A/T- and G/C-centric κB DNA. We speculate that p50 homodimer can bind the GGGGGCTCCC sequence with GGGGGC T CCCXX (two half sites are depicted with underline, X = any nucleotide). Therefore, the p50 homodimer prefers G/C rich κB sites so long as they have the A/T bp at the center. Despite their significant sequence homology, the molecular basis of NF-κB proteins’ differences remains unknown.

The three-dimensional structure of the p52:RelB heterodimer bound to a κB DNA is known. No such structure of the p52:ReA heterodimer exists. Given the structural similarity of different NF-κB:DNA complexes, it would be surprising if p52 subunit of the heterodimer bound DNA any differently. p50:RelA heterodimer binds consensus or near consensus κB sites (such as Ig-κB or MHC-κB sites) with high affinity. Both p50 and p52 homodimers also bind these with high affinities. Both RelA and cRelA homodimers bind similar sites with significantly lower affinity (∼10-fold less). This is probably due to the adaptability of p50 and p52 to the DNA sequence and structural variation. This suggests that p50 and p52 are the dominant partners when they bind DNA as heterodimers with RelA, cRel or RelB in dictating DNA binding affinity. It should also be noted that equilibrium binding affinity does not truly reflect their transcriptional potential.

## Transcriptional Regulation by Non-DNA Binding

The DNA binding transcription factors (TFs) are known for attaching directly and specifically to their DNA response elements, allowing them to control gene expression (Leung et al., 2004; Wan et al., 2007; Mulero et al., 2018; Mulero et al., 2019). This was their exclusive function until new regulatory roles for these factors emerged. TFs can also operate as cofactors to regulate gene expression by not directly binding to DNA but by binding to other TFs that are directly attached to their DNA response elements. In gene regulation, most, if not all, DNA binding TFs use both modes. Several studies have shown that one-third to half of NF-κB-bound active promoters and enhancers are devoid of κB motif (Martone et al., 2003; Lim et al., 2007; Wang J. et al., 2012; Zhao et al., 2014; Inukai et al., 2017). Alternative motifs that specifically recruit other TFs have been identified, suggesting that other TFs recruit NF-κB to these sites through protein-protein interaction. p52 also regulates gene expression using both modalities—by directly binding to the κB DNA and by indirectly binding to non-κB DNA via another TFs through protein-protein interactions. p53 and ETS are two well-studied systems in which the cofactor function of p52 is apparent (Figure 4A).

It was reported that p52 cooperates with p53 to promote the expression of three well-known p53 target genes: p21, DR5 and PUMA (Schumm et al., 2006). The authors arrived at this conclusion after demonstrating that a p52 mutant lacking the capacity to bind κB DNA was recruited to the promoters of the p53 response elements alongside p53. This DNA-binding-independent transcriptional activity of p52 is similar to the ‘cofactor’ function of other transcription factors. The cofactor function of p52 was supported later by Tergaonkar and colleagues. They discovered that p52 supports recruitment of ETS1 to the −146C > T mutant promoter of the telomerase *TERT* gene (Li et al., 2015). A binding site for ETS1 is created by a single C to T bp change at position −146 position. Indeed, the X-ray structure of the p52:ETS1:DNA complex reveals that p52 has no contact with the DNA (Xu et al., 2018). The dimerization domain of p52 interacts with ETS1. p52 activates the *TERT* promoter by at least two mechanisms: first, p52 relieves auto-inhibition of ETS1 DNA binding by directly engaging at a site in the ETS1 DNA binding domain where the inhibitory domain interacts; and second, it increases the affinity of the ETS1:DNA complex. The affinity of the p52:ETS1 complex is low. That is, the binary complex is weak, implying that the cofactor only transiently interacts with ETS1. This might be a common mechanism of cofactor action where weak and transient interaction between a TF and a cofactor is sufficient for elucidating function. Although other NF-κB subunits can form complexes with ETS1, p52 appears to impose most stable interaction. Four of the five residues of p52 that are engaged in forming the ETS1 complex are unique to p52 implying the specificity of this weak complex.

Transcriptional repression of phosphodiesterase 4 (PDE4) by p52 is a curious case as reported recently (Zhang et al., 2019). This report has shown a p52-ChIP DNA fragment sequence that contains no κB consensus or even a half-consensus site. Therefore, it is possible that p52 acts as a cofactor, as in the case of the mutant *TERT* promoter. Surprisingly, however, the genome sequence reveals a consensus κB site (GGGGA T TCCC) close to the ChIPed site. This A/T-centric κB site could potentially recruit p52 and Bcl3 to repress transcription.

p52 has recently been shown to act as a cofactor of CTCF, PU.1, IRF4 and ETS. In lymphoblastoid B cell lines (LCLs), ChIP assay revealed association of p52 with genome fragments that contain no κB sites but response elements of ETS, PU.1, IRF4 and CTCF suggesting p52 might act as a cofactor of these DNA binding TFs for the regulation of genes in *cis* with these targets (Zhao et al., 2014). Since ETS1 has been shown to use p52 for the expression of the TERT gene, it is highly likely that ETS family transcription factors use p52 to support DNA binding. Further studies are required to evaluate the authenticity of cofactor function of p52 at these non-κB sites through interaction with these TFs.

## Cooperation of p52 With RelB and Bcl3

Like all proteins in a cell, p52 interacts with hundreds of different proteins. RelB and Bcl3 are the two proteins that stand out among them (Zhang X. et al., 2007; De Silva et al., 2016) (Figure 4A). RelB is an NF-κB subunit, which is unstable as a monomer and it cannot form a homodimer (Huang et al., 2005; Fusco et al., 2008; Vu et al., 2013). Differences of a few amino acids compared to the other four NF-κB subunits at the subunit interface and the core of the dimerization domain prevent RelB from forming homodimers. It, however, readily forms heterodimers with p52, arguably the most stable of all p52 dimers. The p52:RelB heterodimer is also the most consequential dimer generated through the activation of non-canonical signaling pathways. In addition to interactions through the dimerization domain, RelB and p52 also interact through their N-terminal domains. Interestingly, the RelB subunit of the p52:RelB heterodimer binds κB DNA differently from the RelB subunit of the p50:RelB heterodimer (Moorthy et al., 2007; Fusco et al., 2009). RelB has the ability to bind to a diverse set of κB half sites when the p52 subunit is bound to its cognate half site. It has been reported that p52:RelB heterodimer binds to and activates a unique class of genes such as stromal cell-derived factor 1 (*SDF1*), B lymphocyte chemokine (*BLC*), Epstein-Barr virus-induced molecule-1-ligand chemokine (*ELC*) and secondary lymphoid organ chemokine (*SLC*) (Bonizzi et al., 2004). The p52:RelB heterodimer is involved in the prolonged activation of NF-κB target genes involved in development programs in lymphoid cells (Weih et al., 2001; Yilmaz et al., 2003; Claudio et al., 2006).

The precursor protein, p100, is one defining molecule in shaping the special relationship between p52 and RelB (Basak et al., 2007; Mukherjee et al., 2017). To a considerable extent, p100’s inhibitory function is at least partially RelB-dependent. RelB both stabilizes p100/p52 and blocks p100 processing into p52 (Fusco et al., 2016). These complex and opposing effects on p100/p52 by RelB depends on the NIK activity and concentration. p100 and RelB are synthesized in response to both canonical and non-canonical signaling. Thus, the timing of synthesis, preferential association of RelB with both p100 and its processed product p52 at least partly explain why the p52:RelB is a preferred NF-κB dimer.

The other NF-κB dimer generated by non-canonical signaling is the p52 homodimer. The p52 homodimer, however, is inert as a transcription factor. To a large degree, only when the p52 homodimer is associated with Bcl3 does the homodimer becomes relevant as a transcription factor (Bours et al., 1993). Bcl3 undergoes extensive phosphorylation by several kinases (Nolan et al., 1993; Bundy and McKeithan, 1997). Over 20 phosphorylation sites have been reported in our previous study (Wang et al., 2017). Some of these phosphorylation events induce Bcl3’s degradation (Viatour et al., 2004). At least three of these events are necessary for the Bcl3:p52 complex to act as a transcription factor which regulates transcription by binding to κB DNA. Phosphorylation of S33 is essential for Bcl3’s nuclear localization. Other two phosphorylation sites, S446 in the flexible proline rich C-terminal region and S114 within the ARD are directly involved in rendering Bcl3 a transcriptional coactivator of p52 (Wang et al., 2017). Interestingly, unphosphorylated Bcl3, unp-Bcl3, acts as an inhibitor of p52, similar to the classical IκB proteins: IκBα, IκBβ and IκBε. In other words, p52:unp-Bcl3 is unable to bind κB DNA; p52:p-Bcl3, on the other hand, forms complexes with κB DNAs. And only p52, but not Bcl3, makes direct interactions with κB DNA in the ternary complex. The p52:DNA complex is slightly more stable than the DNA:p52:p-Bcl3 ternary complex. Phosphorylation of Bcl3 apparently induces conformational changes in the protein, allowing it to bind p52 in a way that does not fully inhibit p52’s association with κB DNA. It is noted that, like unp-Bcl3, p-Bcl3 also reduces the stability of p52:DNA complexes; however, unp-Bcl3 is a much more potent inhibitor of the complex than p-Bcl3. These observations can lead to two conclusions: First, as an essential partner Bcl3 enables the p52 homodimer to function as a transcriptional regulator. Second, the resident time of Bcl3 on the p52:DNA complex is a critical factor in determining whether it is transcriptional co-regulator or a p52 inhibitor. The classical IκB protein, IκBβ, has been shown to have dual activities. While IκBβ, primarily functions as an inhibitor of RelA dimers, with specific phosphorylation it acts as transcriptional co-regulator of the RelA homodimer (Rao et al., 2010; Scheibel et al., 2010). The IκB-like inhibitory activity of Bcl3 has not been demonstrated *in vivo*.

## p52 and Disease

**FIGURE 5:**
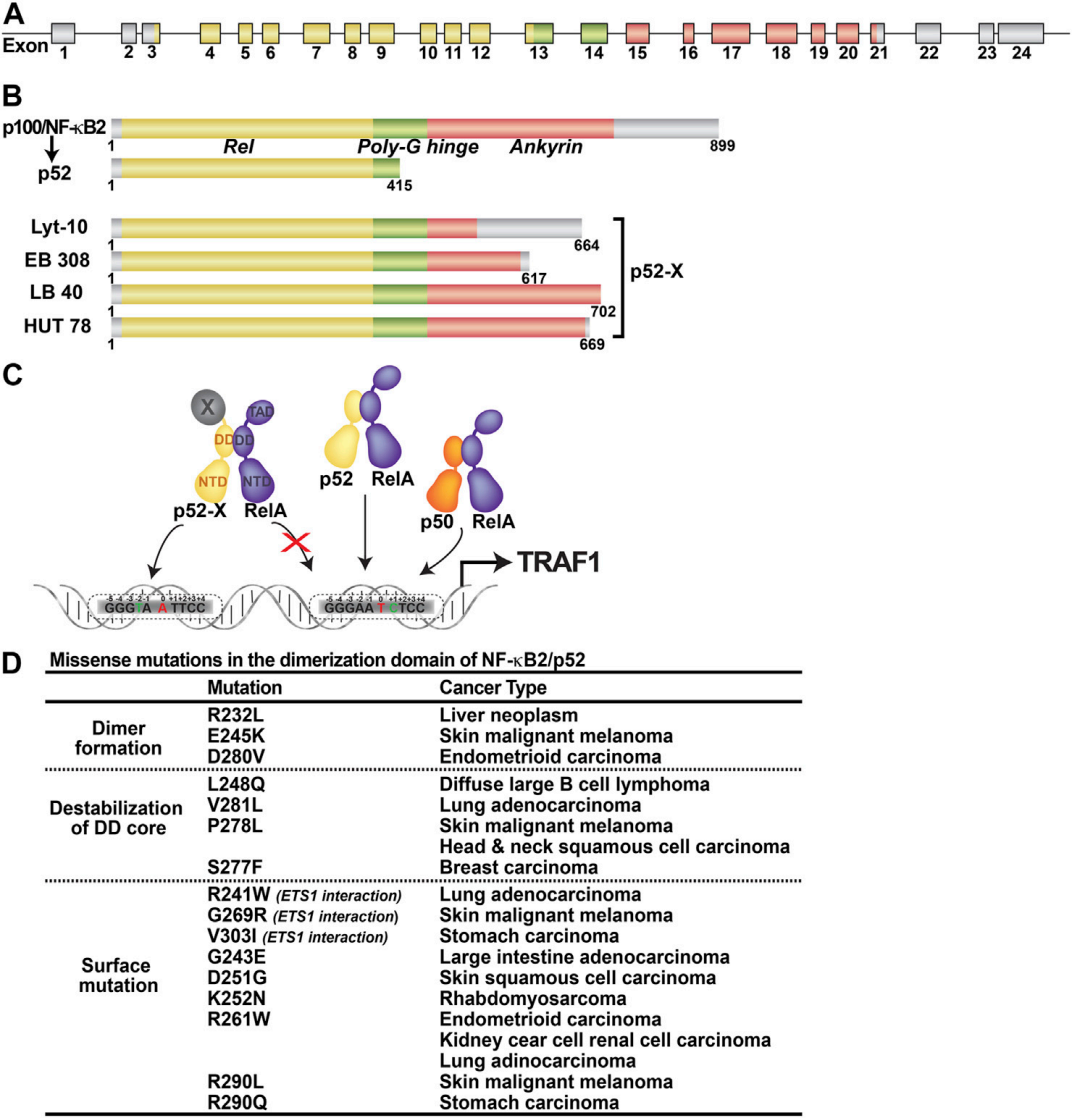
Abnormal products of *nfκb2* gene. **(A)** The *nfκb2* gene is involved in lymphoma-associated chromosomal translocation. Coding exons are represented by color filled boxes. **(B)** Truncated *nfκb2* genes code for C-terminal truncated protein variants lacking different portions of the ankyrin domain, named p52-X. **(C)** Oncogenic p52-X:RelA heterodimer preferentially binds to unfavorable κB site. **(D)** Table showing the missense mutants in the dimerization domain of p52 and the corresponding caner types.

The opposing activities of p100 and p52 make it challenging to properly describe the pathways leading to the functional impairment due to dysregulation of the *nfκb2* gene. As an inhibitor, p100 has the ability to sequester nearly all NF-κB subunits, effectively blocking both canonical and noncanonical functions. Changes in the amount of p52, on the other hand, can alter gene regulation in a variety of ways, including modifying the subunit compositions of NF-κB dimers and affecting promoter binding. The *nfkb2*-knockout mice have defects in secondary lymphoid organ development, peripheral B cell population, humoral responses, and spleen architecture (Caamano et al., 1998; Franzoso et al., 1998). While the absence of p52 results in faulty immune response, the lack of NF-κB inhibitory activity of p100 (Ishikawa et al., 1997) or overexpression of p52, lead to inflammatory diseases and cancer (Dejardin et al., 1995). The over- and under-expression of p52 is the direct result of the p100 processing event. The most obvious reason for the p52-driven disease pathogenesis is the lack of appropriate amounts of p52 and the timing of it. There are several regulators of the p100 processing event and the most critical of which are NIK, IKK1/α, TRAF2, TRAF3, cIAP1 and cIAP2. NIK and IKK1/α are kinases which associate with each other to induce phosphorylation of p100. TRAFs and cIAPs are ubiquitin ligases which act together to maintain low levels of NIK through ubiquitination-mediated degradation. Many additional factors can affect the activities of these primary regulators, thereby affecting p100 processing indirectly. The non-canonical pathway was discovered through characterization of one of the mutants in the form of a mouse strain known as alymphoplasia (aly) mice (Xiao et al., 2001). In these mice, a point mutation in the gene encoding NIK generates a defective protein that cannot associate with IKK1/α resulting in little or no p52. Mutations within the p100 gene understandably impact the processing event. These mutations can be divided into two classes: one class of mutants results in limited or no p52 generation due to defective ubiquitination or NIK/IKK1 phosphorylation; the second class of mutations are truncated p100 resulting from chromosomal translocation or point mutations generating a termination codon within the C-terminal processing inhibitory domain. These truncated mutants process into p52 constitutively (Figures 5A,B). Here we will describe the impact of altered p52 on diseases by highlighting only those derived from chromosomal translocation.

*Nfκb2* gene is rearranged in about 2% of human B- and T-cell leukemias/lymphomas, non-Hodgkin’s Lymphomas, B-cell chronic lymphocytic leukemia (CLL) and multiple myelomas (Neri et al., 1991; Fracchiolla et al., 1993; Migliazza et al., 1994; Thakur et al., 1994; Zhang et al., 1994). Gene rearrangement often results in the production of fusion gene products where the C-terminus of the p52 precursor is shortened and fused to a heterologous gene product which we will refer to as p52-X (Chang et al., 1995) (Figures 5A,B). Constitutive expression of the fusion gene and the nuclear localization of the fusion gene products enforce differential transcriptional programs. These early studies suggested that p52 is an oncogene and even through it cannot drive transformation all by itself, it can do so in collaboration with other factors. Strong supporting evidence comes from a recent study which shows that some cases of *Kras* ^G12D^ driven pancreatic ductal adenocarcinomas (PDAC) progression, the amplification of *nfκb2* gene is required when *Kras* ^G12D^ is not amplified (Mueller et al., 2018). Another study has recently revealed that expression of p52-associated genes is increased in lung adenocarcinomas and correlated with reduced survival (Saxon et al., 2018).

One of the intriguing and yet unresolved issue is whether p52-X, the heterologous protein generated by chromosomal fusion, undergoes constitutive processing to generate p52 or if p52-X is a fully functional transcriptional activator. At least some reports suggest that p52 and p52-X partner with other NF-κB subunits differently and κB DNA binding potentials are different (Zhang B. et al., 2007). p52-X can activate gene expression even when it is not partnered with RelA or Bcl3 suggesting that the ‘X’ segment of p52-X can act as a transcriptional activation domain. Transgenic mice expressing p52-X in lymphocytes develop B cell lymphomas due to the resistance to apoptosis. TRAF1, an anti-apoptotic gene product is highly expressed in these cells. The p52-X:RelA heterodimer induces TRAF1 expression by binding to a specific κB DNA (GGGTA A TTCC) but ignores another κB DNA site located nearby (GGGAA T CTCC) (the central bp is in red color). Although both the κB sites differ from the consensus site by one base pair (underlined), a thymine at −2 position is less acceptable by RelA or p52 compared to a cytosine at +1 position making the later one to be preferred by the p52:RelA or p50:RelA heterodimer (Figure 5C). Why the oncogenic heterodimer prefers an unfavorable site is not known. Overexpression of p52 in lymphocytes of transgenic mice predispose them to inflammatory autoimmune disease characterized by high levels of autoantibodies in the serum. p52, but not p80HT, represses Bim, an antiapoptotic gene, expression, leading to defects in apoptotic processes critical for autoreactive lymphocytes elimination (Wang et al., 2008). The κB site of the Bim promoter is GGGGGCTTCCCCC. This site is favored by the p52 homodimer. It is likely p52 homodimer in association with Bcl3 might be able to repress the Bim expression.

Through large scale sequencing of a range of cancer cell genomes, several missense somatic mutations of NF-κB2/p52 have now been identified (https://cancer.sanger.ac.uk/cosmic). The DNA binding and dimerization domains (DD) are home to many of these missense mutations (Figure 5D). Further research is needed to see if any of these mutations are directly involved in the initiation or progression of cancer. The information collected from structural and biochemical studies, however, can provide some insights into their possible functional anomaly focusing only on the mutations within the DD. Three of the DD mutants (R232L, E245K and D280V) altered interfacial residues where the arginine forms salt bridges with glutamate and aspartate from the opposing monomer. Alanine scanning mutagenesis was used to test the dimerization ability of the corresponding residues in p50. All three mutants showed defect with D302 (D280 of p52) was most defective in the p50 homodimer formation. We expect all three p52 mutants to be dimerization deficient. Another three mutants, V281L, P278L and L248Q, are expected to affect dimerization through destabilization of the core of the p52 DD since their side chains are projected into the hydrophobic core. Three additional surface mutants, R241W, G269R and V303I, are expected to affect ETS1 interaction since they either directly contact ETS1 or are positioned near the p52-ETS1 interface. We are tempted to speculate that some of the mutants may bring new dimer partners that are not seen in normal cells. Several mutants within the DNA binding domain are expected to affect DNA binding either directly or indirectly by destabilizing the DNA binding domain.

## Conclusion

In this review, we highlighted the importance of a few sequence variations in p52 compared to the four other members in imparting distinctive functional properties. We looked beyond the apparently functionally important residues, such as residues at dimer or protein-DNA interfaces, since they are nearly identical in all cases. Distinctive dimerization propensity is likely dictated by the hydrophobic residues of the domain core, which professes the side chain dynamics of interfacial residues. Similarly, residues away from the protein-DNA interface also play a vital role in the DNA recognition process in two ways-by directly affecting the conformation of the interfacial residues and by interacting with other proteins.
